# Machine learning versus crop growth models: an ally, not a rival

**DOI:** 10.1093/aobpla/plac061

**Published:** 2022-12-01

**Authors:** Ningyi Zhang, Xiaohan Zhou, Mengzhen Kang, Bao-Gang Hu, Ep Heuvelink, Leo F M Marcelis

**Affiliations:** Horticulture and Product Physiology, Department of Plant Sciences, Wageningen University, PO Box 16, 6700 AA Wageningen, The Netherlands; Horticulture and Product Physiology, Department of Plant Sciences, Wageningen University, PO Box 16, 6700 AA Wageningen, The Netherlands; Shanghai Lankuaikei Technology Development Co. Ltd, Shanghai 200120, China; Chinese Academy of Sciences, Institute of Automation, Sate Key Laboratory of Management and Control for Complex Systems (CASIA-MCCS), Beijing 100190, China; Chinese Academy of Sciences, Institute of Automation, National Laboratory of Pattern Recognition (CASIA-NLPR), Beijing 100190, China; Horticulture and Product Physiology, Department of Plant Sciences, Wageningen University, PO Box 16, 6700 AA Wageningen, The Netherlands; Horticulture and Product Physiology, Department of Plant Sciences, Wageningen University, PO Box 16, 6700 AA Wageningen, The Netherlands

**Keywords:** Knowledge, and data, driven modelling, Machine learning, Process, based models, yield prediction

## Abstract

The rapid increases of the global population and climate change pose major challenges to a sustainable production of food to meet consumer demands. Process-based models (PBMs) have long been used in agricultural crop production for predicting yield and understanding the environmental regulation of plant physiological processes and its consequences for crop growth and development. In recent years, with the increasing use of sensor and communication technologies for data acquisition in agriculture, machine learning (ML) has become a popular tool in yield prediction (especially on a large scale) and phenotyping. Both PBMs and ML are frequently used in studies on major challenges in crop production and each has its own advantages and drawbacks. We propose to combine PBMs and ML given their intrinsic complementarity, to develop knowledge- and data-driven modelling (KDDM) with high prediction accuracy as well as good interpretability. Parallel, serial and modular structures are three main modes can be adopted to develop KDDM for agricultural applications. The KDDM approach helps to simplify model parameterization by making use of sensor data and improves the accuracy of yield prediction. Furthermore, the KDDM approach has great potential to expand the boundary of current crop models to allow upscaling towards a farm, regional or global level and downscaling to the gene-to-cell level. The KDDM approach is a promising way of combining simulation models in agriculture with the fast developments in data science while mechanisms of many genetic and physiological processes are still under investigation, especially at the nexus of increasing food production, mitigating climate change and achieving sustainability.

## Teaser

We humans are at the nexus of increasing food production, mitigating climate change and achieving sustainable agriculture. Simulation models are useful tools for dealing with those challenges. Combining process-based models and machine learning to develop a knowledge- and data-driven modelling (KDDM) approach provides the opportunity of taking advantages of both modelling tools. Such a KDDM approach potentially increases the prediction accuracy of current modelling tools used in agriculture while keeping their interpretability at a good level. Parallel, serial and modular structures are three useful structures that can be adopted to develop a KDDM approach for agricultural applications.

## Introduction

Simulation models are useful tools in agricultural research for predicting yield, optimizing crop management, understanding physiological mechanisms, and assisting breeding and cropping system design. Typically, two types of simulation models are used, i.e. process-based models (PBMs) and data-driven models. Process-based models simulate plant growth and development based on underlying physiological mechanisms. Since the 1960s, many process-based crop models have been built, stimulated by the rapidly developing computer science. Most early models (e.g. Simple and Universal Crop Growth Simulator, Decision Support System for Agrotechnology Transfer and Agricultural Production Systems Simulator) are still frequently used and continuously updated and improved (Yin and Van Laar 2005; Holzworth *et al.* 2014; Tovihoudji *et al.* 2019). These models are used not only for predicting yield, but more importantly, to understand the environmental regulation of plant physiological processes and its consequences for crop growth, development and yield. Furthermore, model results may reveal emergent properties resulting from the interactions between individual processes simulated in the model. Such a systematic understanding of the determinants of yield helps with optimizing crop system design and making decisions on crop management practises (e.g. irrigation and fertilization) (Van Ittersum *et al.* 2016; Martinez-Feria *et al.* 2018), as well as providing potential directions for breeding high-yielding cultivars, especially when combining with quantitative trait loci mapping, genome-wide association studies and phenotyping (Rötter *et al.* 2015; Kadam *et al.* 2019; Tsutsumi-Morita *et al.* 2021).

Despite being a powerful tool used in agriculture, PBMs have their own intrinsic drawbacks. First, model parameterization can be a difficult process. Process-based models include many parameters that need to be tuned before doing proper simulations, and some parameters may require a substantial amount of measurements. Second, crop models currently seem to reach their limits, not only for the algorithms used to simulate some of the physiological processes, but also in terms of expanding the boundaries of model simulations. Some processes have long been recognized as hard-to-predict (e.g. development of leaf area index and sink formation), and new algorithms for properly simulating these processes are still under exploration (Yin *et al.* 2021). Additionally, current crop models lack the capacity of downscaling towards the gene-to-cell level (due to unclear molecular mechanisms) and upscaling to the regional and global level (due to significant heterogeneity that cannot be ignored), as well as capturing key responses to environmental acclimation and extreme climate events under global change (Feng *et al.* 2019a; Peng *et al.* 2020; Yin *et al.* 2021).

## Modelling in the Age of Big Data: The Rise of Machine Learning

While process-based modelling is based upon human knowledge, machine learning (ML), which is a data-driven approach, allows computers to learn from experience. The idea starts from Alan Turing’s question ‘Can machines think?’, which was distinguished from the traditional practice where machine systems only operate as manually programmed by humans (Turing 1950). The last 20 years have seen a burst of applying ML in many fields including agriculture. The early agricultural applications of ML can be dated back to the 1990s, with artificial neural networks being the most common algorithm, possibly stimulated by the birth of the back-propagation algorithm which enabled deriving parameter values for complex neural networks (Werbos 1981; Rumelhart *et al.* 1986; Elizondo *et al.* 1994). Capable of regression on complex non-linear relationships, classification and processing unstructured data (e.g. images and sensor data), ML is now widely used for predicting yield, analysing remote sensing data, detecting diseases and weeds, and phenotyping and breeding (Singh *et al.* 2016; Tang *et al.* 2017; San-Blas *et al.* 2020; Van Klompenburg *et al.* 2020). However, ML models encounter the disadvantage of being black-box models, whose structures do not help interpret their parameters or predictions; this makes extrapolation difficult. Moreover, ML models usually need to be trained with a large amount of data.

## Collaboration Between Humans and Machines: Allying PBMs with ML

Both PBMs and ML models have intrinsic drawbacks for model simulations as mentioned above. To develop models with both high prediction accuracy and interpretability, combining human knowledge and ML has long been discussed, and recently interpretable ML marks the new generation of artificial intelligence (Thompson and Kramer 1994; Hu *et al.* 2009; Rudin 2019; Deng *et al.* 2020). In a human–machine collaborating modelling approach, what and how the machines learn should be well-planned according to human knowledge, of which PBMs are one of the best carriers given their capability of summarizing and structuring domain knowledge. In a hybrid modelling approach that combines PBMs and ML (termed the knowledge- and data-driven modelling approach, i.e. the KDDM approach, hereafter), task allocations among ML and PBMs are based on the knowledge embedded in PBMs.

Furthermore, PBMs can lead or restrict how machines learn through four aspects. First, ML algorithms should be selected and modified for certain tasks. For example, to capture the climate effects on crop growth in preceding weeks, specific recurrent neural network architectures such as long short-term memory (LSTM) should be considered for its ability to model relationships between events with long time gaps. Although research has been conducted on employing recurrent neural networks for the prediction of environmental conditions such as rainfall (Fang and Shao 2022; Ouma *et al.* 2022), they have not been further applied for plant growth prediction to the best of our knowledge (Fang and Shao 2022; Ouma *et al.* 2022). Second, for some ML algorithms (e.g. linear regression), equations in PBMs can be used directly as the ML model for which ML learns the parameter values (e.g. intercept and slope) from data. Third, boundaries of variables in PBMs can be embedded into ML algorithms by adding constraints when minimizing the loss function or by choosing certain activation functions in neural networks (e.g. a sigmoid activation function limiting the output value between 0 and 1). While discussed in the field of ML for the improvement of interpretability (Qu and Hu 2011), applications of the second and third points above received limited attentions in agricultural research. Last, PBMs can generate simulation data which largely decrease the demand of experimentally determined training data. This has been applied in autonomous control research where PBMs were used to train the ML for smart decisions (Hemming *et al.* 2019).

## Three KDDM Structures Combining PBM and ML

**Figure 1.**
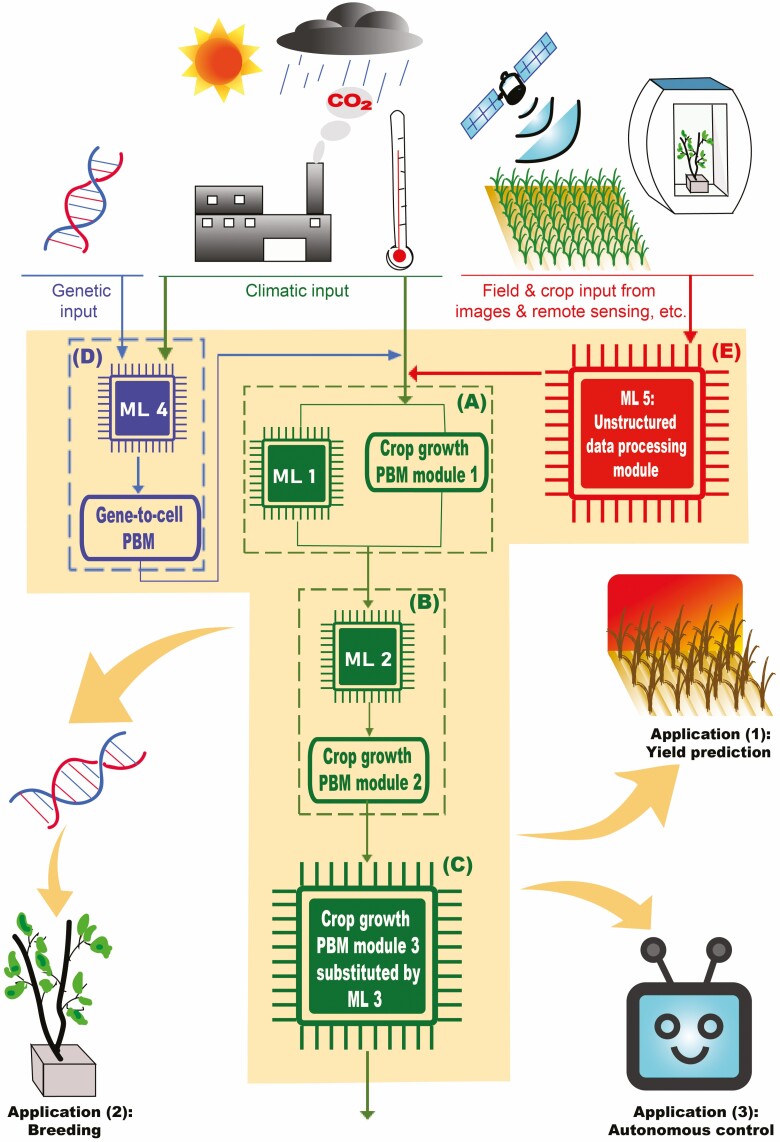
A KDDM approach combining a PBM and ML. The original PBM in the figure has three modules, in which (A) PBM module 1 is combined with ML 1 in a parallel structure, (B) PBM module 2 is combined with ML 2 in a serial structure and (C) PBM module 3 is substituted by ML 3 in a modular structure. The PBM can further combine with (D) ML 4 to allow downscaling towards gene-to-cell level and/or (E) ML 5 to enable the use of unstructured data (e.g. images and remote sensing data). The KDDM approach can be applied for (1) yield prediction under global climate change (e.g. heat stress) and on a regional basis, (2) assisting breeding and (3) developing autonomous growing systems. Thin arrows represent data flow. Thick arrows point to applications.

Modelling an agricultural system involves different patterns of relationships (e.g. linear versus non-linear), different simulation levels (e.g. photosynthesis at leaf level versus yield at crop or production system level) and different types of data (e.g. climate data versus plant data), and thus allocating tasks among PBMs and ML is crucial for taking advantage of both process-based modelling and ML. Here, we select parallel, serial and modular structures for our KDDM approach based on the characteristics of process-based crop models (Fig. 1A–C), and provide examples on task allocation in these structures. Some of these structures exist already for decades, whereas not much attention has been paid to their application in agriculture.

### Parallel structure

In the parallel structure, PBM and ML models have the same input variables, and the KDDM output is either the sum or the multiplication product of the output of PBM and ML models (Thompson and Kramer 1994; Hu *et al.* 2009) (Fig. 1A). For the ‘sum’ structure, ML is trained based on the residual between experimental data and predictions from the PBM. For example, in Fan *et al.* (2015), a combination of ML and the GreenLab model (a PBM taking into account plant 3D architecture) with the ‘sum’ structure increased the prediction accuracy on tomato organ dry weights under different environmental conditions compared with using the GreenLab model only. For the ‘multiplication’ structure, ML is trained based on the quotient obtained by dividing the experimental output values by the predictions from the PBM.

### Serial structure

The serial structure is defined as either running ML model prior to PBM or the reverse (Thompson and Kramer 1994; Hu *et al.* 2009) (Fig. 1B). When ML model is running prior to PBM, the ML model output becomes the parameter or input values of the PBM. This structure can be used to simplify parameterizations, and to convert sensor data to the input of PBM, which is needed in agricultural systems where field data measured by sensors are not exactly the input data for the crop model (see ‘Future Perspectives’). While in the case that PBM is running prior to ML model, the output of PBM is used as input for ML model, so that ML can find the relationship between the output of PBM and the required model output. For example, Kaneko *et al.* (2022) developed a hybrid model with a serial structure: first, a process-based single-leaf photosynthesis model was employed to calculate the value of leaf photosynthetic rate from environmental factors; then an artificial neural network was used to calculate the canopy photosynthetic rate from this single-leaf photosynthetic rate and leaf area index. This hybrid model not only requires a more simple calibration process compared with a process-based canopy photosynthesis model, but also provides a more accurate prediction than using artificial neural network alone to predict canopy photosynthesis from environmental factors, especially when the prediction was conducted outside the range of training data (Kaneko *et al.* 2022).

### Modular structure

With a modular structure, individual PBM modules (e.g. light interception) can be substituted by ML or combined with ML in a parallel or serial structure (Fig. 1A–C). Extra modules (e.g. gene-to-cell module, remote sensing module) can be added to the model to allow downscaling or upscaling of the model and utilizing unstructured data (Fig. 1D and E). To make the most use of both PBMs and ML models and to allow smooth connection between them, changes of variables and data processing might be needed. We provide three examples where the modular structure is or can be adopted.

#### Example 1.

To improve the prediction of wheat yield under extreme climate events (e.g. heat, frost and drought), Feng *et al.* (2019a) developed a hybrid model by incorporating ML in the APSIM model (a PBM predicting crop growth and production with simplified functions describing the effects of extreme climate events). In the hybrid model, Feng *et al.* (2019a) substituted APSIM’s module of predicting wheat yield from total biomass and development stage with ML. Their results showed that the hybrid model improved prediction accuracy by 33% compared to APSIM alone. It needs to be noted that extra data processing is needed between the output of development stage from APSIM and the ML module, where Feng *et al.* (2019a) counted the duration of extreme climate events in each development stage. Such data processing shows how human knowledge can play a role in connecting PBM and ML modules, beyond selecting the hybrid structure and developing PBMs. We recommend considering such knowledge-based data processing rather than simply using the PBM output as the ML model input, in order to take the most advantage of both process-based modelling and ML.

#### Example 2.

Fan *et al.* (2015) tested the modular structure by substituting GreenLab’s module of predicting potential biomass from climate factors by a neural network, as a comparison with the parallel structure. While GreenLab only considered global radiation and temperature as inputs and ignored other factors such as CO_2_ concentration and humidity, Fan *et al.* (2015) tested several ML modules with different climate factors as inputs. It is not surprising that in their study the modular structure where the ML module included more climate factors outperformed the parallel structure. This comparison between modular and parallel structures shows the advantage of modular structure where the re-selection of variables can improve the simulation accuracy.

#### Example 3.

Remote sensing, through which data can be derived without on-site observations, can be added as a module when modelling an agricultural system. For the remote sensing module, ML and PBMs can be combined in two ways. First, ML runs after remote sensing (serial structure) for tasks such as processing unstructured data (e.g. images, satellite signals) and classifying remote sensing results (e.g. ML links vegetation indices derived by remote sensing to water stress categories; Romero *et al.* 2018). Second, ML can be combined with physical models inside the remote sensing module to boost accuracy (Yuan *et al.* 2020).

## Limitations

Specific limitations should be noted when combining PBMs and ML. First, a large amount of data is needed for training ML. Second, the three structures of the KDDM approach only combine PBM with ML model; however, the ML algorithm needs to be selected carefully to ensure its capability of simulating the target process. For example, the random forest algorithm is frequently used to simulate the relationships between yield and climate variables. However, random forest has limited capability to capture the impact of the past climate on future growth and yield; i.e., climate variables at one time point not only affect crop growth at this time point but also have prolonged effects in coming days. For dealing with such relationships, LSTM neural networks generally outperform random forest (Schwalbert *et al.* 2020). When combining ML with PBMs for dynamic relationships, the time structure (e.g. how many time steps in the future are influenced by a change at the current time step) should be considered in both models. Third, adding a black-box model to a PBM brings extra uncertainties to the model that cannot be avoided, and replacing part of the PBM potentially hampers the interpretability of the original PBM. Therefore, in the modular structure, the modules that will be replaced by ML need to be evaluated carefully with expert knowledge. Generally, we recommend to only replace the modules in a PBM that use simple empirical relationships and assumptions without considering mechanisms underlying the simulated process, and in such cases human knowledge on the specific process needs to be embedded into ML. Modules with solid underlying physiological mechanisms should be kept in the original PBM.

## Future Perspectives

The KDDM approach that combines the strength of PBMs and ML potentially increases the prediction accuracy of current modelling tools used in agriculture while keeping their interpretability at a good level. In a broad sense, the KDDM approach would be closer to human-like artificial intelligence, considering that we humans learn from both prior knowledge and experience in our daily life. For agricultural applications, the KDDM approach can be envisioned at three integration levels.

First, at the crop and cropping system level, the KDDM approach simplifies model parameterization and improves the prediction accuracy. Machine learning algorithms are developed to link sensor data with plant physiological properties. For example, ML is used for predicting crop nitrogen content from hyperspectral data, obtaining plant architectural traits and detecting stress from phenotypic data, and predicting leaf photosynthetic parameters from leaf reflectance spectra (Heckmann *et al.* 2017; Ziamtsov and Navlakha 2019; Berger *et al.* 2020; Feng *et al.* 2020). These plant information (e.g. nitrogen content and architectural traits) are all parameters needed in crop models but require laborious and time-consuming measurements when determined in a conventional way. By combining PBMs and ML, the input parameters of the hybrid model can be acquired via advanced sensor technologies, thus simplify the parameterization of the hybrid model while still keeping all physiological processes of PBMs. Additionally, combining PBMs and ML also simplifies the acquisition process of training data for ML used in phenotyping, since synthetic data generated by PBMs can be used as training data for ML (Lobet *et al.* 2017; Ubbens *et al.* 2018). Moreover, the KDDM approach is useful for improving the accuracy of yield prediction under global climate change (Fig. 1). The mechanisms of crop responses to climate change are still under study, which makes mechanistic simulation of these processes difficult. These individual processes can be simulated using a data-driven approach based on historical climate and yield data, and then be integrated into a PBM or substitute relevant modules in a PBM (Feng *et al.* 2019a).

Second, by incorporating ML, the KDDM approach potentially allows upscaling the simulation level of a crop model towards a farm, regional or global level, with the potential to predict the effects of heterogeneity. For example, ML can classify the farms in a region with the help of remote sensing, and combining such ML with a PBM that predicts crop growth on a single farm level. While ML has been used for classification tasks such as soil mapping, it has not been combined with crop models for large-scale crop growth prediction to the best of our knowledge (Forkuor *et al.* 2017). At farm-, regional- or global-level KDDM can assist system optimization and decision support (e.g. designing autonomous control algorithms, risk assessment for insurance application, and weed and stress detection for crop management decisions) (Gao *et al.* 2018; Feng *et al.* 2019b; Hemming *et al.* 2019; Schwalbert *et al.* 2020; Li *et al.* 2021). For example, KDDM can generate large amounts of simulated data for a wide range of conditions, especially plant data (e.g. biomass) which is impossible to collect by conventional experiments in a short period given this is both time- and labour-consuming. Also, these simulation data can be used to develop autonomous control systems for crop production (e.g. optimizing greenhouse climate settings without human interference; Hemming *et al.* 2019) (Fig. 1). This could further provide directions for sensor development based on the most important crop parameters or environmental data needed for the autonomous control system.

Third, the KDDM approach could downscale to the gene-to-cell level to allow predicting from genotype to phenotype and assisting breeding (Fig. 1). Processes that currently can hardly be simulated mechanistically can be data-driven, while simulations in other modules (e.g. from organ to plant) can be process-based (Kadam *et al.* 2019). This provides an opportunity to link the gene-level data to crop growth and development processes, given that currently it is impossible to simulate genotype to phenotype using a full mechanistic model. For example, ML has been used to link genetic marker data with phenological parameters, and by further combining with a crop model, the hybrid approach is able to predict the performance of a new genotype in a new environment (Chen *et al.* 2020).

In conclusion, the KDDM approach that combines the strength of both PBMs and ML is a useful tool at different integration levels. Currently, we humans are at the nexus of increasing food production, mitigating climate change and achieving sustainable agriculture. The KDDM approach is a promising way of using simulation models for agricultural applications, given that data science is fast developing, whereas mechanisms of many processes in yield production are currently still under exploration. Parallel, serial and modular structures are three useful structures for combining PBMs and ML. Knowledge-based data processing is recommended when transferring PBM output into ML input, or vise versa. We expect that the three structures for combining PBMs and ML demonstrated in this paper will boost the application of such a hybrid modelling approach in agricultural science.

## Data Availability

No data were generated for this study.

## Notes

**Plants, Ecosystems & Climate. Chief Editor:** Mary Heskel
